# The Effects of Transitions in Caregiving and Changes in Social Participation on Old Adults’ Depressive Symptoms

**DOI:** 10.1093/geroni/igaa057.1140

**Published:** 2020-12-16

**Authors:** Xinyi Zhao, Huiying Liu, Quan Zhang

**Affiliations:** 1 Peking University, Beijing, China; 2 Central South University, Changsha, Hunan, China; 3 Peking University, Beijing, China, China

## Abstract

Objectives: This study aimed to investigate the effects of transitioning into spousal caregiving, changes in social participation, and their interactions on depressive symptoms among community dwelling old adults over time. Methods: The samples included old adults who were non-caregivers at 2011 baseline of China Health and Retirement Longitudinal Study (CHARLS) and joined the follow-up surveys in 2013 and 2015. Generalized estimating equations (GEE) was used to analyze the effects of caregiving transitions (transitioning into low-intensity or high-intensity caregivers versus the non-caregivers) and changes in social engagement on the depressive symptoms over time. Results: The results showed that old adults who transitioned into spousal caregiving over a 4-year period reported more depressive symptoms than those remained non-caregivers. Old adults who continued or increased social participation reported fewer depressive symptoms than those without social participation. Individuals who continued social participation during the transitions into high-intensity caregiving showed less severe elevated depressive symptoms than their counterparts who did not engage in social participation. Conclusion: The results highlighted that continuous social participation might be a protective factor for old adults against negative psychological outcomes during the transition to high-intensive caregiving.

